# Cortical Dynamics during the Preparation of Antisaccadic and Prosaccadic Eye Movements in Humans in a Gap Paradigm

**DOI:** 10.1371/journal.pone.0063751

**Published:** 2013-05-09

**Authors:** Isabel Cordones, Carlos M. Gómez, Miguel Escudero

**Affiliations:** 1 Neuroscience and Behavior Group, Departamento de Fisiología, Facultad de Biología, Universidad de Sevilla, Seville, Spain; 2 Human Psychobiology Lab, Departamento de Psicología Experimental, Facultad de Psicología, Universidad de Sevilla, Seville, Spain; Hospital General Dr. Manuel Gea González, Mexico

## Abstract

To compare the cortical dynamics of different oculomotor tasks, EEG and eye movements were recorded in 21 volunteers. Using a comprehensive approach, subjects were asked to perform saccadic tasks, which included a saccadic eye movement to a peripheral target (prosaccadic), a movement to the opposite side (antisaccadic), or maintain the gaze fixed (no-go). In mixed trials, prosaccadic, antisaccadic and no-go tasks were indicated by a color square (S1) present for 1900–2500 ms (instructive period). S1 disappeared for 370 ms (gap) and a black dot at 8 deg at right or left indicated the beginning of the task. Reaction times, amplitude of eye movements and number of errors were greatest in antisaccadic tasks, suggesting a greater difficulty. The EEG showed a contingent negativity variation (CNV) that increased progressively along the instructive period and suddenly during the gap: higher in antisaccadic, followed by prosaccadic and no-go tasks. Principal component analysis (PCA) disentangled fronto-central and occipital CNV-related and fronto-central gap-related components. The instructive period was characterized by fronto-central and occipital beta desynchronization (ERD) higher in antisaccadic than in no-go and parieto-occipital alpha synchronization higher in no-go than in antisaccadic tasks. During the gap, parieto-occipital beta and alpha ERD were higher in antisaccadic compared to no-go. The gap was further characterized by a fronto-central increase of inter-trial coherence in theta: highest during antisaccadic, followed by prosaccadic and no-go tasks. This phase locking in theta was also accompanied by theta ERS, which was significantly higher in antisaccadic than in the other two tasks. In PCA of spectral power two main components had dynamics similar to those extracted from voltage data, suggesting cross-frequency coupling. These results suggest that the more difficult saccadic tasks are associated with top-down control mediated by frontal cortex, while simpler tasks rely more on bottom-up control mediated by posterior cortices.

## Introduction

In antisaccadic tasks, when a visual target appears on one side of the screen, subjects must move their eyes to the opposite side. The antisaccadic task allows evaluation of the capacity to inhibit reflexive saccades and produce voluntary saccades [1]. Antisaccadic eye movements are more difficult tasks to perform, which increases response latency and the number of errors compared to prosaccadic tasks. The antisaccadic task is thought to involve the inhibition of the more prevalent prosaccadic response and the activation of a more forced response. Given the greater difficulty of this task with respect to prosaccadic tasks, a heightened control is needed for its success.

A number of approaches have been taken to explore differences in the preparation and execution of saccades during prosaccadic and antisaccadic tasks, including recording the activity of single neurons, functional magnetic resonance imaging (fMRI) and lesion studies. Single-neuron recordings in the superior colliculus (SC) and the frontal eye field (FEF) during the instruction period have shown increased activity of fixation-related neurons and decreased activity of saccade-related neurons during antisaccadic compared to prosaccadic trials. This activity pattern explains the longer reaction times (RT) on antisaccadic trials [2], [3].

By fMRI, the preparatory period has been previously related with a higher activity in FEF, supplementary eye field (SEF), dorsolateral prefrontal cortex (DLPFC), anterior cingulate cortex (ACC), supplementary motor area (SMA) and intraparietal sulcus during the antisaccadic in comparison with prosaccadic tasks [4], [5], [6], [7], [8]. In this sense, lesion experiments and pathological conditions involving DLPFC and ACC seem to induce an increased number of errors during the antisaccadic task [9].

Although considerable effort has been made to describe prosaccadic and antisaccadic tasks processing during preparatory and response periods, technical characteristics of the fMRI impose important limitations on the study of temporal dynamics. EEG-derived measures such as ERPs and time-frequency analysis provide higher temporal resolution. It is well known from EEG studies that when antisaccades or prosaccades are preceded by a cue indicating the type of task, a contingent negative variation (CNV) appears during the preparatory period. This CNV has a higher amplitude at fronto-central regions during antisaccadic than during prosaccadic tasks [10], [11], [12]. This increase in CNV amplitude during the antisaccadic task is in good agreement with fMRI findings of increased activity in the same regions during antisaccadic compared to prosaccadic tasks [8].

Another technique to examine cortical dynamics associated with the processing of stimulus-response is event-related frequency analysis. Major frequency changes have been observed in the EEG during preparatory periods for motor responses in which a decrease of spectral power or event-related desynchronization (ERD) of the mu rhythm occurs [13], [14]. In other respects, the use of these high-resolution techniques allows the study of certain cognitive processes associated with sensory preparation and motor preparation and execution. For example, an ERD of beta rhythm would infer a motor preparation process [13]; a posterior ERD of alpha, anticipation of the imperative stimulus; and an anterior mu ERD, preparation for the response [15]. The role of the working memory in the antisaccadic and prosaccadic tasks could be evaluated by means of the theta and alpha event-related synchronization (ERS), which have been proposed as a possible carrier frequency for working memory processes [16]. The working memory role is particularly important in experiments mixing antisaccadic and prosaccadic trials in the same block [17], given that the instructional value of the cue must be remembered throughout the preparatory period. This is the case for the present study.

An additional complexity in this kind of experiments is when the antisaccadic and prosaccadic tasks are accompanied by a gap period -a temporal interval between switching off the central fixation point and switching on the peripheral target- which induces a decrease in RT [18], [19], [20]. This reduction of the RT associated with the temporal gap is independent of the type of motor response and has been demonstrated for saccadic eye movements and manual responses [20], [21], [22], [23]. Two different hypotheses have been proposed to account for this effect: first, the gap acts as a warning, which would include the motor preparatory processes [24], [25]; and second, attention is disengaged from the central fixation stimulus, which is assumed to be an automatic process that occurs prior to the peripheral target during the gap period [19], [20], [26]. Growing evidence has supported motor preparation as an additional factor in the gap effect [26].

During the gap period, a frontal gap-related negativity has been found in the ERP [27], [28], [29], [30]. This frontal negative component, which appears as a rapid deflection following the visual offset-evoked potentials triggered by switching off the central fixation point, was proposed as indexing preparatory processes in the frontal cortex. The scalp distribution and the relationship to facilitation in RT suggested that the frontal negativity could represent the electrophysiological index of the activation of premotor, supplementary motor, and/or motor cortex.

In addition to the antisaccadic and prosaccadic tasks, the present experiment included a no-go task, in which the subjects must ignore the imperative stimulus and inhibit their response. No-go task could be useful as a control during the preparatory period because no movement is prepared but memory load is similar to the other tasks.

The aim of the present study was to identify differences in cortical activities during the instruction and gap periods in antisaccadic, prosaccadic and no-go tasks. Comparisons of saccadic parameters and RT suggested that antisaccades were more difficult and required higher attention to be performed than prosaccades and no-go. In parallel, during the instructive and gap periods, a higher frontal activity was found in antisaccadic than in prosaccadic and no-go tasks. This increased frontal activation was strongest during the gap period indicating that a higher top-down control must be expected in the more demanding tasks, particularly at the moment in which a precise timing is required.

## Materials and Methods

### Participants

Twenty-one subjects (6 males and 15 females) aged between 21 and 29 years (mean = 23.5±2.8 years) were recruited from the University of Seville student community, and gave their written and informed consent to participate. They reported having no neurological diseases and normal or corrected-to-normal vision. Experiments were approved by the Ethics Committee of the University of Seville (10/24/2007) and adhered to the principles of the Declaration of Helsinki (1964).

### Behavioral paradigm

**Figure 1:**
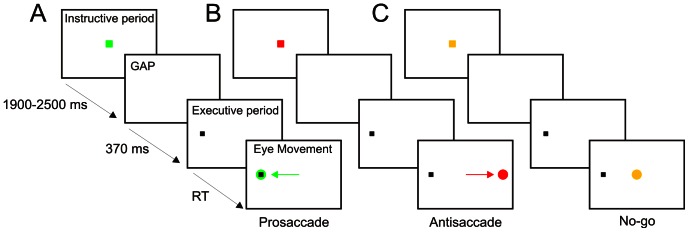
Experimental design. The experimental session involved 240 trials of intermixed prosaccadic (A), antisaccadic (B) and no-go (C) trials. Each trial comprised an instructive period in which a central color square (S1) on a white background identified the type of task trial (red for antisaccade, green for prosaccade or yellow for no-go) during a variable time of 1900–2500 ms. The central color square was switched off during 370 ms (gap period) and a peripheral black dot target (S2) appeared, randomly located at 8 deg on the left or right side, indicating the executive period. Subjects were instructed to execute eye movements with the shortest possible reaction time (RT) after S2. Arrows indicate the correct direction of the eye movement in each task and the color circle the appropriate final position of the eyes.

Participants were seated 68 cm in front of a CRT monitor on which the stimuli were displayed. They were asked to avoid head movements during the experimental session. Each session consisted of 240 trials of randomly intermixed prosaccadic, antisaccadic and no-go tasks (80 trials per task). In each trial, the subject maintained a fixed gaze on a colored square (0.6×0.6°) (S1) at the center of a white background screen (Fig. 1). The color of this square served as a cue: green instructed subjects to perform a saccade directed to an eccentric black dot (prosaccadic task, Fig. 1A); red indicated a saccade to the side opposite where the black dot appeared (antisaccadic task, Fig. 1B); yellow directed them to avoid any eye movement (no-go task, Fig. 1C). The time between switching the S1 stimulus on and off (instructive period) was randomized between 1900 and 2500 ms. After the instructive period, the cue disappeared during 370 ms (gap period) before an eccentric dot (S2, the beginning of the executive period) subtending a visual angle of 0.3 deg appeared in the horizontal meridian at 8 deg on the left or the right side of the screen in a pseudorandom order. The eccentric dot was present during 1000 ms.

### Eye position and EEG recordings

Visual stimuli were generated using Eevoke software (ANT, Holland) on a PC with a CRT monitor (refresh rate of 80 Hz and resolution of 1024×768). Trigger information from Eevoke was sent to the EEG recording system. Eye movements in the horizontal plane were recorded by infrared video-oculography (Chronos 3D Binocular Eye Tracker, Chronos Vision GmbH, Germany). This system sampled the position of both eyes at 400 Hz. Data from eye positions were automatically calculated by the EyeTracking software (Chronos Vision GmbH, Germany) and the position of the eyes were digital-to-analog converted (2 ms delay) and recorded throughout the EEG amplifier as external signals. EEG activities were collected from 64 scalp tin electrodes mounted in a cap using the International 10–20 system. Voltages from electrodes were measured with respect to an average reference. Impedance was maintained below 5 KOhms for each electrode. EEG signals and eye movements were recorded through a full-band DC amplifier (ASA-lab EEG/ERP system, ANT, Holland) at a sampling rate of 1024 Hz.

### Detection, parameters and statistical analysis of saccadic eye movements

Saccadic eye movements were automatically detected by a script programmed in Matlab 2008a (MathWorks Inc., MA, USA) using eye velocity threshold [31]. For each saccadic eye movement, the beginning, ending, peak velocity, timing to peak velocity, amplitude and duration were analyzed. The RTs were calculated as the period between the arrival of each eccentric target and the moment in which the eye reached its maximum velocity during the saccade. Only correct saccades were analyzed. Errors in direction, anticipations (<100 ms) or delays (>400 ms) with respect to the eccentric target, and saccades not related to the task were not considered. Percentage of error was calculated for each task and an arcsine transformation was applied for statistical comparisons.

RTs were calculated for prosaccadic and antisaccadic tasks for rightward and leftward-directed saccades. Mean RTs were compared per saccadic directions and tasks. Errors between antisaccadic and prosaccadic tasks were compared for each type of error. Statistical analysis comparisons of saccadic eye movement parameters, errors and RTs in the different tasks were carried out by one-way analysis of variance. When the normality condition failed, Wilcoxon test was applied; when homogeneity of variance was not achieved, a student t-test for related measures was performed.

### Statistical analysis of EEG activities

EEG recordings were analyzed with EEGLAB rev.10.0.0.0b toolbox [32] using Matlab 2008a (MathWorks Inc., MA, USA) software package. To eliminate AC power line interference and blink artifacts in the EEG, an independent components analysis [33], [34], [35] was performed. Criteria for determining these components were their scalp map distribution, time course and spectral power. Thus, the eye blink artifact component showed a frontal location, coincided with blinking in the recording of eye movements and had low frequency in the power spectrum. These components were discarded and the EEG signal reconstructed. For the analysis of EEG, data were segmented in intervals of 2100 ms, from 1800 before to 300 ms after the visual eccentric target. The baseline was corrected by subtracting for each channel the mean voltage level in the first 100 ms interval of the window. For each task and subject, data were averaged to obtain ERP by using S2 as a trigger. The ERPs obtained during the three tasks were statistically compared using permutations. Comparisons were considered as significant when probability (p) values were below 0.05. A false discovery rate for multiple comparisons [36] was applied using EEGlab toolbox.

A time-frequency EEG analysis was performed trial-by-trial using Hanning-windowed sinusoidal wavelets at 1.5 cycles (lowest) to 7.5 cycles (highest). Changes in event-related dynamics of the EEG spectral power were studied using the event-related spectral perturbation (ERSP) index [37]. ERSP quantifies the mean change in spectral power (dB) from the baseline at different latencies and frequencies with respect to the event. Event-locked EEG phase coherence was computed by ITC, analogous to the “phase locking factor” [38]. Significance thresholds for ERSP and ITC were calculated by a bootstrap distribution (p<0.01), extracted randomly from the baseline data (100 first ms of each epoch) and applied 200 times [39]. Additionally, the ERSP and ITC of the different experimental conditions were statistically compared by permutation analysis (p<0.05), taking into account false discovery rate for multiple comparisons.

In parallel, a principal component analysis (PCA) was applied to ERP and ERSP data. The PCA allows extraction of the total variance of the data as a few components that can be identified as sources of variance in the empirical data [40]. In this particular data set, the main objective was trying to disentangle different components for ERP and ERSP that could separate neural activities during the instructive and gap periods. Although PCA analysis allows the use of several types of axes rotations and all procedures are mathematically correct, we used the more parsimonious non-rotated orthogonal approach.

The PCA of ERP data was carried out on a matrix containing time columns and rows for voltage, electrodes, subjects and conditions. The matrix was similar for the PCA of the ERSP, replacing voltage with ERSP frequencies (3 to 30 in 0.5 hertz bin). The topographies of the components were obtained by averaging the component scores for the extracted components. For the ERSP matrix, only loading factors were taken into account. Given the high complexity of the component scores, analysis of those results would require a separate study.

Specific applications of the PCA method that are relevant to understanding the dynamics of CNV and brain rhythms during the instructive and gap period include the following:

1. PCA analysis in the voltage domain would permit separation of brain dynamics during the gap period from those of the previous instructive period.
2. The topographical representation of the component scores in the time domain would help to disentangle the relative contribution of different components in the instructive period (namely the CNV) and the gap-related negativity.
3. In the time-frequency domain, PCA would identify similarities, if any, between the dynamic of the components extracted by time frequency analysis and of those obtained from the voltage domain. In case of similarities, a frequency-coupling between voltage and the power of brain oscillations could be argued.

## Results

### Behavioral response

**Figure 2:**
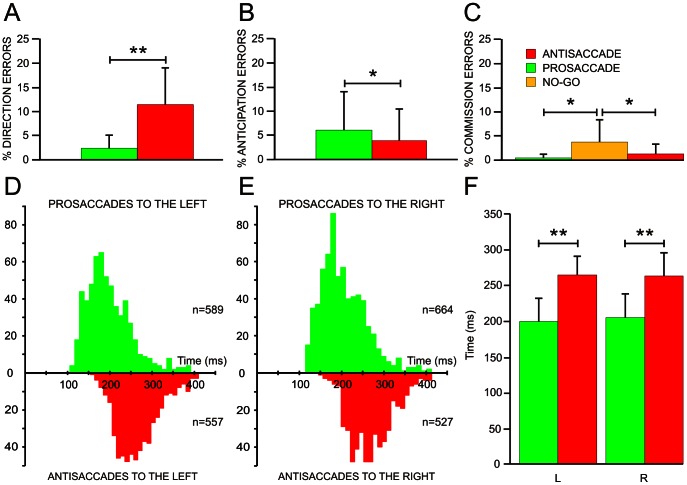
Errors of execution and saccadic latencies of prosaccadic and antisaccadic eye movements. Percentage of direction (A), anticipation (B) and commission (C) errors during prosaccadic (green), antisaccadic (red) and no-go (yellow) tasks. In D and E, histograms of saccadic latencies for prosaccades (up, green) and antisaccades (down, red) directed to the left (D) and to the right (E). In F, mean saccadic latencies and standard deviation for prosaccadic and antisaccadic eye movements directed to the left (L) and to the right (R). The asterisks indicate significance of differences: *p<0.05, **p<0.01.

All subjects performed the tasks skillfully, providing a mean of 87.9±7.7% (mean +/− SD) of correct responses, although different tasks showed different degrees of difficulty. The percentages of total errors during the experiments were 19.2±11.6, 13.2±10.1 and 3.9±4.6% for antisaccadic, prosaccadic and no-go tasks, respectively (one-way ANOVA, *F*(2,60) = 19.59, p<0.01). Main differences were due to errors in direction in antisaccadic (11.4±7.7%) and prosaccadic (2.3±2.8%) tasks (p<0.01, Wilcoxon test) (Fig. 2A). Other major errors were due to anticipated responses(<100 ms), which were significantly higher in prosaccadic (6.0±8.2%) than in antisaccadic (3.9±6.6%) tasks (p<0.05, Wilcoxon test) (Fig. 2B). Finally, significant differences in errors (p<0.05, Wilcoxon test) were due to execution of saccades when the subject was instructed to not perform eye movements in the no-go task (3.7±4.6%) in comparison to the lack of eye movement during prosaccadic (0.5±0.7%) and antisaccadic (1.3±2.0%) tasks (Fig. 2C).

Figures 2D and 2E show prosaccadic and antisaccadic latency distributions to the left and to the right side. Saccadic latencies were shorter in prosaccadic (202.5±31.9 ms) than in antisaccadic (263.8±28.7 ms) tasks, regardless whether eye movements were directed to the left or to the right (One-way ANOVA, *F*(1,40) = 42.90, p<0.01) (Fig. 2F). Moreover, with respect to the size of saccadic eye movements, mean amplitude of antisaccades (8.3±1.2 deg) was significantly greater than in prosaccadic (7.5±0.4 deg, t-test, *t*(20) = −3.19, p<0.01 tasks. Taken together, the differences in number and type of errors and in response time suggest that differences between the three tasks could be explained by a higher degree of difficulty to prepare and execute antisaccades compared to prosaccades, and the same for prosaccades compared to no-go responses. Furthermore, the greatest amplitude in antisaccadic compared to prosaccadic tasks could suggest excessive motor preparation, which could also be related to the higher difficulty of the antisaccadic task.

### EEG voltage activities during the instructive and gap periods

**Figure 3:**
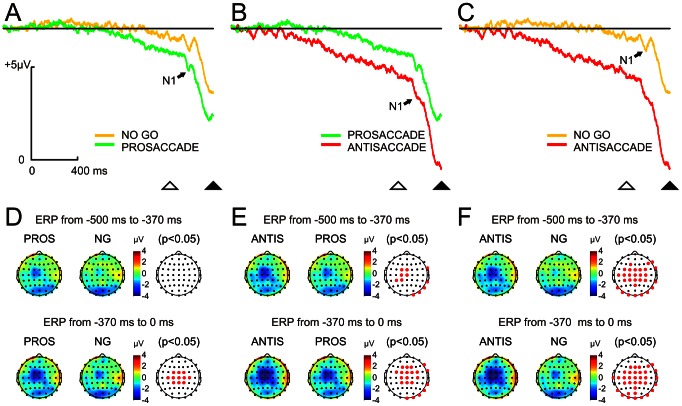
Event-related potentials (ERP) and statistical comparison of scalp maps during the preparatory period. From A–C, ERP comparison between each pair of tasks at the electrode FCz during the preparatory period. Color traces indicate the ERP for each type of task (red for antisaccade, green for prosaccade and yellow for no-go). During the instructive period (while S1 was present), a progressive negative potential was developing. During the gap period, this negative potential increased abruptly. The black horizontal line denotes the baseline value. This negativity was greater during prosaccadic than no-go (A), during antisaccadic than prosaccadic (B) and during antisaccadic than no-go (C) tasks. The offset of S1 (open triangle) and the onset of S2 (filled triangle) are indicated. Please note the visual evoked potentials (N1) triggered by the offset of S1 (black arrows). Calibration and voltage polarity for A–C is indicated in A. D–F show in the first two columns the topographical voltage distribution and in the third the statistical comparison between the same pairs of tasks shown in A–C, during the last 500 ms of the instructive (top row) and gap (bottom row) periods. Scalp distribution of voltage negativity was mainly located fronto-centrally. During the instructive period, significant differences in negativity were exclusively found between antisaccadic versus prosaccadic and no-go tasks. During the gap period, significant differences were found between the three tasks. Red points indicate electrodes with statistical differences (p<0.05).

During the instructive period, a negative potential was slowly developing on the scalp in all three tasks. This negativity, which was maximal at FCz, increased steadily until the beginning of the gap period. Figure 3 shows the grand average ERP at FCz in no-go and prosaccadic (Fig.3A), prosaccadic and antisaccadic (Fig. 3B) and no-go and antisaccadic (Fig. 3C) tasks, triggered by S2 (black arrowhead in Fig. 3A–C). The extinction of S1 (white arrowhead in Fig. 3A–C) induced a visual-offset evoked potential -whose component N1 was clearly visible on the traces- and a sudden increase of the negative potential. During the gap period, maximum negativities were reached in FCz at 290 ms, 330 ms and 350 ms after S1 extinction in prosaccadic, antisaccadic and no-go tasks, respectively.

The negativity during both the instructive and gap periods displayed a very similar fronto-central and posterior topography (Fig. 3D–F). The first two columns in the figure 3D–F display scalp voltage distributions during the instructive (top row) and the gap (bottom row) periods in the indicated time windows. The third column in the same figure shows statistical comparison between each pair of the indicated tasks. During the instructive period, significant differences (p<0.05, permutation analysis and false discovery rate for multiple comparisons) were obtained between antisaccadic and prosaccadic tasks in the fronto-central (more negative in anti- than in the prosaccadic task) and in the right temporo-occipital areas (more negative in pro- than in the antisaccadic task) (Fig. 3E). The comparison between antisaccadic and no-go conditions also showed significant differences in fronto-central (more negative in antisaccadic than in the no-go task), and occipito-temporal areas (more negative in no-go than in the antisaccadic task) (Fig. 3F). No differences were found in scalp voltage between prosaccadic and no-go tasks preceding the gap (Fig. 3D).

During the gap period, differences in scalp voltages were located fronto-centrally and were significant for the comparisons between the three tasks (Fig. 3D–F, bottom). Consequently, the ERP was more negative in antisaccadic than in prosaccadic, and in prosaccadic than in no-go tasks. Moreover, there were significant differences in antisaccadic with respect to prosaccadic (Fig. 3E) and no-go (Fig.3F) tasks in occipital regions (more negative in pro and no-go than in antisaccadic conditions).

**Figure 4:**
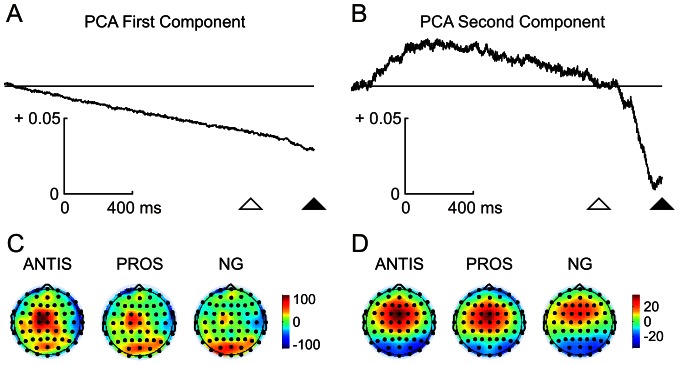
Principal component analysis (PCA) of the event-related potentials during the preparatory and gap periods. The first PCA component (A) displays a trend line during all the preparatory period whereas the second (B) shows positivity during the instructive period and a sharp negativity during the gap period. These two PCA components explained 79% and 7% of the variance, respectively. Scalp component scores for the first and second components of the PCA during antisaccadic (ANTIS), prosaccadic (PROS) and no-go (NG) tasks are shown in C and D, respectively. Please note that the occipital component is only present in the first component.

The similitude in scalp voltage distribution during the instructive and gap periods was appealing. As the gap induces deep changes in response time, it seems reasonable to expect more differences between these two periods. To test the possibility that some signals during the instructive period would continue during the gap period and mask gap-related signals, a spatiotemporal decomposition of the ERP by PCA was carried out (Fig. 4). The PCA of the ERP responses yielded two main components that explained 79% and 7% of the total variance, respectively. The PCA's first component displayed a linear downward trend in temporal behavior during the instructive and gap periods (Fig. 4A), whereas the second component displayed a positivity during the instructive period and a sharp negativity during the gap period (Fig. 4B). The topography of the component scores for the first component showed a central distribution whose amplitude values were highest in antisaccadic, mid-level in prosaccadic and lowest in no-go tasks. Moreover, the first component displayed an occipito-parietal topography with an inverse amplitude pattern for the three tasks compared to central sites (Fig. 4C). The second component exclusively displayed a fronto-central topography with an amplitude pattern relationship to each task (Fig. 4D) similar to the first component's central distribution.

**Figure 5:**
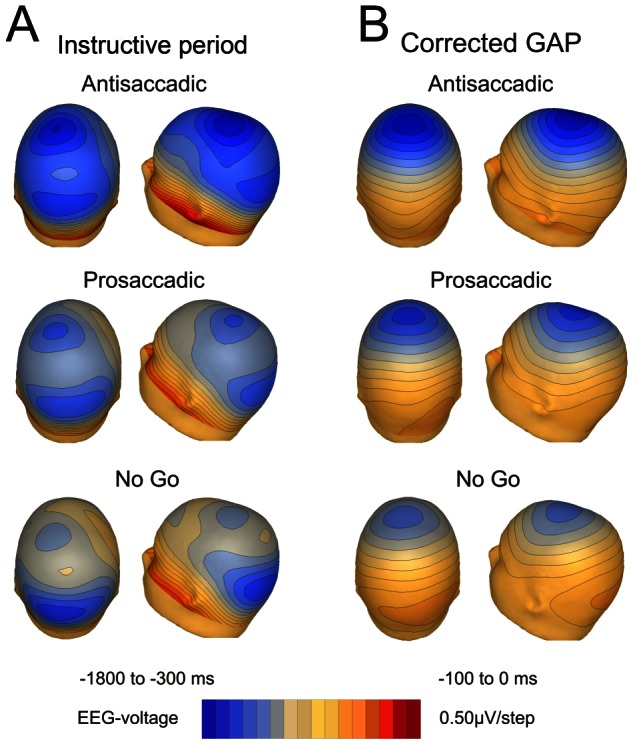
Voltage topographies. The scalp voltage distribution during the instructive period (A) displayed fronto-central and posterior negativity. The topography during the last 100 ms of the gap period, after subtracting the trend line during the instructive period, showed the fronto-central but not the occipital component (B).

As the PCA suggested the existence of linear negative potential during the instructive period that continued during the gap, the slope of the trend line was corrected for each subject and electrode and the voltage map recalculated. The slope value of the ERP average was calculated for each subject in each electrode during the time period from −1800 to −300 ms. With these slopes, a theoretical line was generated and subtracted from the ERP average in the whole window (from −1800 to 0 ms), generating the corrected averaged signal. The baseline was calculated at the beginning of the gap period and the inter-subject grand average recalculated. The Figure 5A shows the scalp voltage during the instructive period with the central and occipital activities described above. Figure 5B shows the scalp voltage during the gap period after correction; only negativity at fronto-central, but not at posterior regions, was present in all three tasks.

These results indicate that: i) during the instructive period, all three tasks induce a central negativity that intensifies sharply during the gap period in fronto-central electrodes; ii) this fronto-central negativity is greater in antisaccadic than prosaccadic and in prosaccadic than in no-go tasks; and iii) the occipital negativity is established during the instructive period and although it continues during the gap it is not intensified.

### EEG frequency activities during the instructive and gap periods

**Figure 6:**
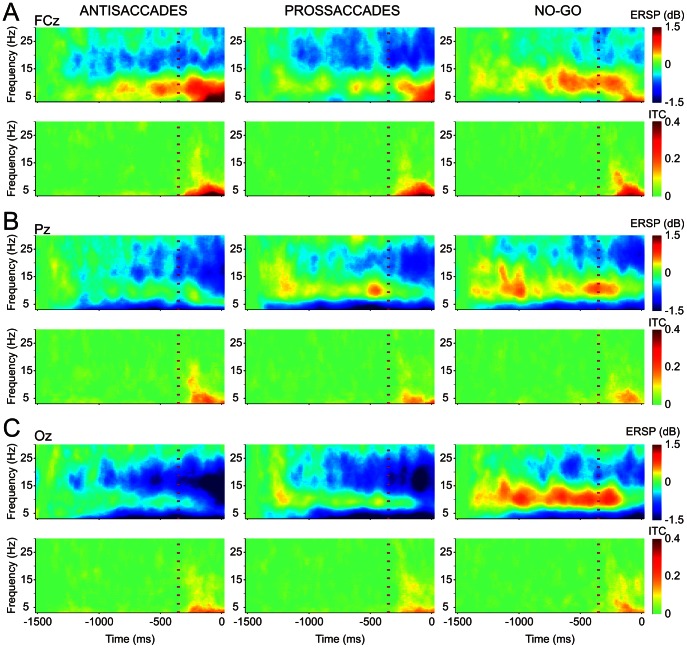
Event-related spectral power (ERSP) and inter-trial coherence (ITC) during the preparatory period. The figure shows the time-frequency analysis at FCz (A), Pz (B) and Oz (C) for antisaccadic (ANTIS), prosaccadic (PROS) and no-go (NG) tasks. For A–C, significant (bootstrap p<0.01) differences (non-green pixels) in ERSP (first row) and ITC (second row) with respect to the 100 first ms are shown. The vertical dashed red line indicates the onset of the gap period. For ERSP analysis, warm color indicates even-related synchronization and cool color event-related desynchronization. For ITC, warm color indicates increase in phase coherence.

Brain dynamic activities were also evaluated by ERSP and ITC analyses during all three tasks, revealing differential activities at the fronto-central and parieto-occipital cortex. Figure 6 shows the ERSP (Fig. 6A–C above) and ITC (Fig. 6A–C below) analyses during the three tasks at FCz (A), Pz (B) and Oz (C). At parieto-occipital electrodes, this analysis showed theta (3–8 Hz) and beta (13–22 Hz) ERD during the instructive and gap periods in the three tasks and, mainly in the no-go task, alpha (8–13 Hz) ERS during the instructive and at the beginning of the gap periods. The gap (vertical dashed red line in Fig. 6) induced, at parieto-occipital electrodes, an ERD from 3–22 Hz that was more evident in the antisaccadic task. During the gap, there was also a fronto-central theta ERS in the three tasks (Fig. 6A). The ITC analyses showed significant increases in theta and alpha at fronto-central level during the gap period in all three tasks.

**Figure 7:**
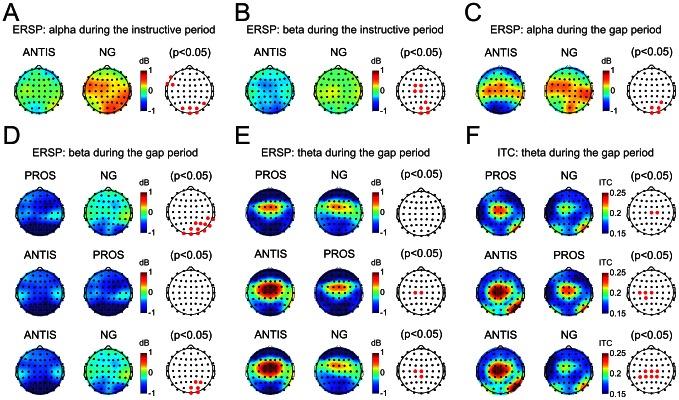
Topographical distribution of theta, alpha and beta rhythm activities during the instructive and gap periods. A and B show scalp distribution of event-related spectral power (ERSP) in alpha and beta bands, respectively, during the instructive period. Comparisons between tasks (third column) showed a significant event-related synchronization (ERS) in no-go (NG) with respect to antisaccadic (ANTIS) task in the alpha band (A), and an event-related desynchronization (ERD) for antisaccadic compared to no-go task in the beta band (B). C–F show the scalp distribution of ERSP and inter-trial phase coherence (ITC) during the last 270 ms of the gap period. Parieto-occipital regions were characterized by a significant alpha ERD in antisaccadic with respect to no-go tasks (C) and a beta ERD higher in antisaccadic and prosaccadic than in no-go tasks (D). Fronto-central activities were depicted by theta ERS and ITC. Theta ERS was higher in antisaccadic than prosaccadic and no-go tasks (E). Theta ITC task comparisons showed best phase locking in antisaccadic followed by prosaccadic and no-go tasks (F). Red points in the third column indicate significant differences (p<0.05). Calibration bars are indicated.

To disclose spectral differences between the tasks, a paired comparative analysis was performed (Fig. 7). During the instructive period, differences in ERSP were found in alpha and beta bands. In alpha, there was a parieto-occipital ERS which was significantly higher (p<0.05) in no-go than in the antisaccadic task (Fig. 7A). In beta, by contrast, there was a fronto-central and parieto-occipital ERD, and this was significantly higher in antisaccadic than no-go tasks (Fig. 7B).

In the alpha band, the parieto-occipital ERS observed during the instructive period switched to an ERD during the gap. This ERD was greater in antisaccadic than in no-go tasks (p<0.05) (Fig. 7C). Similarly, during the gap period, there was an ERD in beta at parieto-occipital electrodes that was significantly greater in prosaccadic and antisaccadic than in no-go tasks (Fig.7D). These results indicate that alpha and beta ERD also seem to be related to the degree of task difficulty and the consequent attention required to perform it. During the gap period, significant differences between tasks were found in the theta band in both spectral power and coherence. The ERSP analysis showed a significant fronto-central theta ERS in antisaccadic compared to prosaccadic and no-go tasks, but not between prosaccadic and no-go tasks, indicating that antisaccadic preparation require a higher recruitment of neurons (Fig. 7E). By contrast, differences in theta ITC between the three tasks were significant (p<0.05) at fronto-central levels (Fig. 7F), meaning that better neuron synchronization is required to prepare for an antisaccadic than for a prosaccadic and for a prosaccadic than for a no-go response. This phase locking behavior in theta indicates that neural synchronization induced by the gap is a fundamental preparatory activity for the imminent arrival of the S2. Differences in the intensity of the theta ITC furthermore suggest that theta is also modulated by the degree of difficulty of the task being performed.

**Figure 8:**
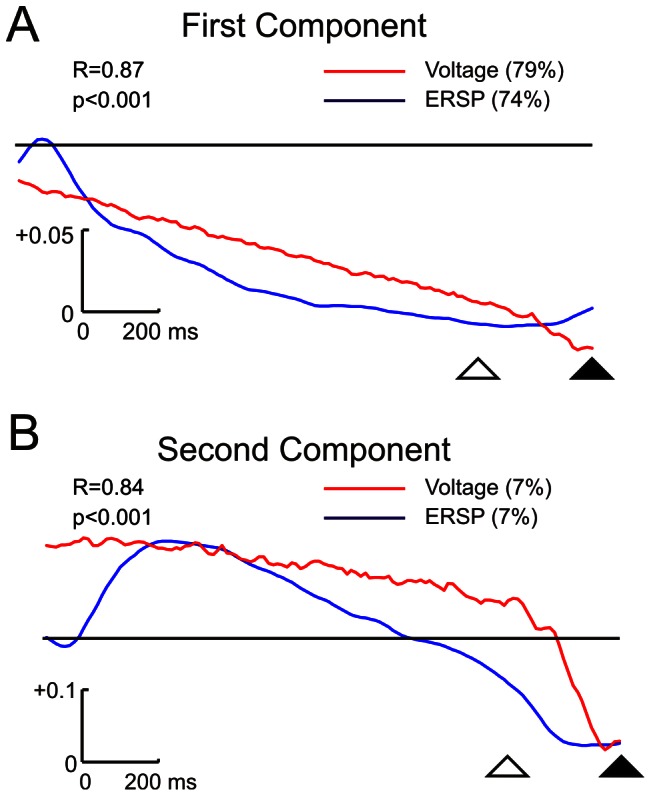
Principal component analysis loading factors obtained from voltage and event-related spectral power (ERSP) values. A and B show the temporal evolution of the loading factors of the first and second components, respectively. The explained variance is indicated between parentheses. The correlation coefficient (R) between voltage (red line) and ERSP (blue line) loading factor components and its statistical significance (p) are shown in each figure. The open and filled triangles indicate the end of the instructive and gap periods, respectively. Calibrations are indicated.

A PCA was applied to ERSP data obtained from wavelet analysis. This frequency-spatio-temporal decomposition yielded two main components, explaining 74% and 7% of the total variance. The first PCA component (Fig. 8A) displayed a decay along the instructive period, whereas the second (Fig. 8B) showed positivity during the instructive period and negativity during the gap. Comparisons of these two PCA components with those obtained from ERP data revealed similar profiles whose correlation coefficients were significant for the first (r = 0.87, p<0.001) (Fig. 8A) and the second (r = 0.84, p<0.001) (Fig 8B) PCA components. These results indicate that not only voltage but also the frequency of EEG signal differentiate the instructive and the gap periods, suggesting a frequency coupling between brain rhythms and voltage negativities.

## Discussion

Complex behavioral paradigms that incorporate different types of tasks, such as prosaccadic, antisaccadic and no-go conditions, and include a gap period between the extinction of the cue and the target offer a very useful approach to the study of cortical dynamics because they include motor and sensory preparation, working memory, attention and executive motor processes. Analysis of behavior, ERP, ERSP, ITC and topographical analysis would reveal the brain signal computations specifically related to each of these processes. The present report refers only to the instructive and the gap periods.

### Behavioral results

It is well known that antisaccadic responses show an increased latency and number of errors compared to prosaccadic response [1], [41]. Accordingly, the present results show that latency and number of errors were higher in antisaccadic than in prosaccadic tasks and that errors were mainly due to direction mistakes. The number of errors in the antisaccadic task increases when a concurrent working memory task is performed [42]. The need to keep a given operative rule in working memory is particularly obvious in an experimental design in which the type of trial indicated by the cue is updated in each trial. Latency differences may be related to the extra time needed to inhibit the reflexive glance toward the target and to command the eye movement to the opposite side in the antisaccadic task [1]. Errors of execution in which subjects performed a movement during no-go or did not produce any eye movement during saccadic tasks were also observed. These two types of errors are probably due to confusion about the type of trial in which the subject was embedded, producing response in no-go and no response in prosaccadic and antisaccadic tasks. Taking into consideration the response timing, number and type of errors, and the effect of experimental design complexity, it could be concluded that there is an increasing order of difficulty from no-go to prosaccadic and antisaccadic tasks.

### Event-related potentials during the instructive period

In these experiments, the preparatory period included an instructive period –in which a cue color indicating the type of task was present– and a gap period –in which the cue disappeared 370 ms before the appearance of S2. During these preparation times, different processes such as rule retention by working memory, timing, and sensory and motor preparation must be expected to be active. However, each process probably develops at different times and can change its relative importance depending on the type and difficulty of the task.

In ERP analysis, the instructive period was characterized by a progressive slow negative potential, located in the fronto-central and posterior regions of the scalp. This negative potential can be considered a preparatory CNV. The frontal component probably corresponds to the classical motor preparation subcomponent of CNV [43] whereas the posterior is probably related to sensory anticipation [44], [45]. These components presented different amplitudes between the different tasks.

During the instructive period, the negativity in frontal sites was significantly higher in antisaccadic tasks, followed by prosaccadic and no-go tasks. A reverse pattern was found in posterior sites, with higher amplitude in prosaccadic and no-go than in antisaccadic tasks. An increased CNV in central and pre-central regions in antisaccadic versus prosaccadic tasks has been previously described [10], [11], [12], but the reverse pattern for occipital negativity observed here constitutes a new result. The task-related opposite pattern of negativity in posterior and anterior regions was also revealed by the topography of the first PCA component, which exhibited the same arrangement (Fig. 4). These results seems to indicate that the activity during the instructive period is biased to frontal sites as the difficulty of the task increases and more control is needed for producing an accurate response. The preparatory period has been previously related with a higher activity of FEF, SEF, DLPFC, ACC, SMA and intraparietal sulcus by fMRI experiments during the antisaccadic task [4], [5], [6], [7], [8]. In a fMRI study with a paradigm very similar to that of the present report, Brown et al. (2006) showed an increase in the BOLD signal during the instructive period in antisaccadic with respect to no-go tasks in left and right FEF and left caudal precuneus [46]. The comparison of prosaccadic and no-go tasks yielded an increased activation in the left precuneus during the prosaccadic condition. The differences between fMRI and the present study could be attributed to the fact that the EEG measures the brain activity related to changes in different rhythms of activity that can contribute in different directions and intensities to brain metabolism.

### Event-related potentials during the gap period

During the gap period, a sudden increase in negativity occurred at fronto-central and parieto-occipital regions. The PCA results can disentangle this sharp increase in negativity during the gap from the slow negativity during the instructive period. The PCA showed two main components, one with fronto-central and parieto-occipital distributions which would be related to the CNV during the instructive period and extended into the gap period, and a fronto-central component whose temporal dynamics and topography explain the variance associated with gap-related negativity [29].

The continuation of CNV activity during the gap period explains why there is a parieto-occipital voltage activity very similar to that observed during the instructive period. However, fronto-central negativity during the gap was greater than during the instructive period, indicating extra fronto-central activity during the gap period.

Voltage activities during the instructive and gap periods in antisaccadic and prosaccadic tasks would have sources in areas described in fMRI for general and motor related preparation. The fronto-central localization strongly suggests that the RT facilitation attributed to the gap compared to the overlap paradigms is probably due to motor preparation, as suggested by previous studies using behavioral and ERP data [22], [24], [25], [27], [28], [30], [47].

The gap-related negativity was roughly located over the SMA and/or dorsal ACC. This increased negativity in the SMA could explain the increased activity in the superior colliculus observed during the gap period [48], [49], [50]. Extensive connections between the SMA and the superior colliculus have been well established [51], and this pathway would be implicated in the shortest RTs of saccades preceded by a gap period. However, motor preparation could not completely explain the gap-related negativity in antisaccades (which require inhibition of prevalent responses) and no-go conditions. Given that gap-related negativity must account for motor preparation, inhibition of the prevalent responses and probably timing, it could be related to the more cognitive concept of attentional control [52] and monitoring of the ongoing processes, which would be higher in antisaccadic than prosaccadic and no-go conditions.. The dorsal ACC have a role in top-down control of oculomotor regions, including the FEF [53], and an increased number of errors in antisaccadic tasks is related to lower activation in dorsal ACC [54]. Therefore we can suggest a role for dorsal ACC during the gap period to exert cognitive control in the proposed tasks; as more cognitive control is needed, more neural activity is associated with it, i.e. in the antisaccadic task in the present experiments.

### Time-frequency results

During the instructive period, there was an increase in alpha power in the parieto-occipital cortex (significantly lower in antisaccadic than no-go tasks) and a decrease in beta power in the fronto-central and parieto-occipital (greater and slightly more frontal in antisaccadic than no-go tasks). Decreases in ERS in the alpha band has been related to complex tasks and effort in attention [13], suggesting that antisaccadic task execution is more demanding than the others. Motor preparation could be observed throughout the power in the beta band. Beta ERD has been previously related to the functional state of the somatosensory and motor cortex [14], [55], [56]. In this sense, it is interesting to note that beta ERD was smallest in the no-go task, i.e., when no motor response was required.

In the present experiments, there was a broadband theta, alpha and beta ERD in parieto-occipital sites during the gap period. Broadband power reduction has also been demonstrated in the late phase of the CNV [14], [57]. The alpha ERD was significantly greater in antisaccadic than no-go tasks. The other comparisons (anti- vs. prosaccadic and prosaccadic vs. no-go) did not yield significant differences. Beta ERD was significantly greater in prosaccadic and antisaccadic than in no-go tasks. These differences in activity during the gap period could be related to the higher difficulty, attention and sensorimotor preparation required by the antisaccadic task. In the theta band, there was an ERS at fronto-central level with respect to baseline for all three tasks. Differences were found between the antisaccadic task and prosaccadic and no-go tasks that could be related to the high attention required [58], [59] to prepare the more demanding [60] antisaccadic task. The increase in theta power at fronto-central sites was also accompanied by increased ITC. Theta band would also serve as the carrier for working memory [16] to retrieve the rule [61], [62], [63], [64], [65] needed to invoke the appropriate response. Following the definition of Botvinick et al. (2001) of cognitive control [52], this increase in theta oscillation would be related to the contextual information, in this case the type of current trial. This increase in fronto-central theta ITC was significantly higher in antisaccadic than in prosaccadic and no-go tasks, indicating that the so-called gap negativity –reflecting control processes– is partially generated by theta ITC.

### PCA results

Comparison of the loading factors of the PCA extracted from voltage and ERSP during the instructive and gap periods presented very similar temporal dynamics. This indicates a functional linkage between voltage and spectral power, as previously suggested by the broadband decrease in power that occurs during the CNV period [14]. Funderud et al. (2012) obtained a similar result in a go/no-go experiment [57] and suggested that the CNV would reflect the synchronization of a low frequency rhythm [66], and could trigger a cross-frequency coupling with the classical brain rhythms. The similar dynamics of the PCA components obtained from CNV, gap-related negativity and ERSP clearly support this suggestion.

Present results suggest that when a cue indicates tasks of increased difficulty, sensory-motor preparation includes increased fronto-central CNV and beta ERD and decreased alpha ERS parieto-occipital activity with respect to tasks with lesser cognitive demands. On the other hand, the presence of a gap before the target acts as a pre-saccadic trigger signal producing a fronto-central negativity mainly related with theta ERS and ITC. This gap-related negativity, which was accompanied by beta and alpha ERD, was probably related to top-down control of the cognitive and sensory-motor processes needed to produce an accurate response.
